# Search for computational modules in the *C. elegans *brain

**DOI:** 10.1186/1741-7007-2-25

**Published:** 2004-12-02

**Authors:** Markus Reigl, Uri Alon, Dmitri B Chklovskii

**Affiliations:** 1 Cold Spring Harbor Laboratory, Cold Spring Harbor, NY 11724, USA; 2 Department of Molecular Cell Biology and Department of Physics of Complex Systems, Weizmann Institute of Science, Rehovot, 76100, Israel

## Abstract

**Background:**

Does the *C. elegans *nervous system contain multi-neuron computational modules that perform stereotypical functions? We attempt to answer this question by searching for recurring multi-neuron inter-connectivity patterns in the *C. elegans *nervous system's wiring diagram.

**Results:**

Our statistical analysis reveals that some inter-connectivity patterns containing two, three and four (but not five) neurons are significantly over-represented relative to the expectations based on the statistics of smaller inter-connectivity patterns.

**Conclusions:**

Over-represented patterns (or motifs) are candidates for computational modules that may perform stereotypical functions in the *C. elegans *nervous system. These modules may appear in other species and need to be investigated further.

## Background

There is little doubt that neurons are elementary building blocks of the nervous system [1]. It is less clear, however, whether multi-neuron modules (smaller than invertebrate ganglia or vertebrate nuclei and cortical columns) can be meaningfully defined, either anatomically [2] or physiologically [3]. The existence of such multi-neuron modules would greatly simplify the description of nervous system structure and function. An example of such simplification can be found in electrical engineering. An electronic circuit is often represented in terms of modules such as operational amplifiers, logical gates and memory registers rather than as a wiring diagram showing each transistor, resistor and diode. However, unlike electrical engineers who designed these modules themselves, neurobiologists did not design the brain, and evolution rarely leaves records of its experimentation. Therefore, if multi-neuron modules have indeed evolved they need to be discovered.

In this paper, we search for anatomically defined multi-neuron modules in the *Caenorhabditis elegans *nervous system. We choose *C. elegans *as a model organism because its wiring diagram is largely known, including the identities of all 302 neurons and most synapses between them [4-6]. Our approach follows the reasoning developed previously in the context of gene regulation and other networks [7,8]. If a certain multi-neuron module performs some stereotypical function it may appear in the nervous system repeatedly. Therefore, search for multi-neuron connectivity patterns that appear more often than by "chance" (compared with the expectations as defined below) may yield these multi-neuron modules. Of course, there may be functionally important modules that appear infrequently and would be missed by our analysis. In the electronic circuit analogy, our approach would discover logical gates in a processor wiring diagram but not a rectifier in a power supply, which is essential but appears only once.

To search for *N*-neuron modules, we sort all *N*-neuron combinations into classes defined by their inter-connectivity pattern and count the number of combinations in each class. By comparing these counts with the mean counts from random networks, constructed based on our expectations, we detect significantly over-represented patterns, or motifs. In order to avoid assigning significance to a *N*-neuron pattern just because it contains *N-1*-neuron motifs we incorporate the *N-1*-neuron statistics into the expectations used to search for *N*-neuron motifs [8]. To do this, we perform our search sequentially, by starting with doublets (or neuronal pairs, *N *= 2) and then increasing the number *N *of neurons included in the pattern sequentially up to quintuplets (*N *= 5).

We look for motifs in the wiring diagram of the *C. elegans *nerve ring (a large fraction of the nervous system) assembled in two datasets [6]. Datasets 1 and 2 were obtained from serial-sections electron microscopic (EM) reconstructions of two different animals [4]; for details see Methods. The datasets contain the numbers of synapses formed in a subset of *C. elegans *neurons. Two given neurons may be connected by more than one synapse, which we call the multiplicity of connection. However, the small size of the dataset compels us to use the binary representation of these connections (connected or unconnected). In order to obtain binary connectivity matrices, we threshold the multiplicity of connections at various values Θ: Pairs having less than Θ synapses are considered unconnected while those having at least Θ synapses are considered connected. Such procedure is justified because more than a single synaptic contact may be necessary for an observable physiological effect of one neuron on another. Since we do not know the physiologically relevant count of synapses, we repeat our calculation for 1 ≤ Θ ≤ 7.

Unfortunately, datasets 1 and 2 contain a caveat of synaptic ambiguities, which arises from the limitations of EM in *C. elegans*. When one pre-synaptic neuron makes contact with two adjacent processes of different neurons (send_joint in Durbin notation [6]), it is not known which of these processes acts as a post-synaptic terminal; both might be involved. We address this ambiguity by performing our analysis in two ways. In the main text we present the results obtained on the datasets that include both send and send_joint synaptic connections. We repeated the analysis on the datasets where send_joint synapses were split equally between the two potential post-synaptic partners. Specifically, we calculated multiplicity of connections by adding send_joint synapses at 50% synaptic strength. In the limit of high multiplicity, this is equivalent to assigning the post-synaptic neuron by chance. We find essentially the same results for this connectivity dataset (see Supplementary Information [Additional file 1]).

## Results

### Bi-directionally connected doublets (*N *= 2) are over-represented

**Figure 1:**
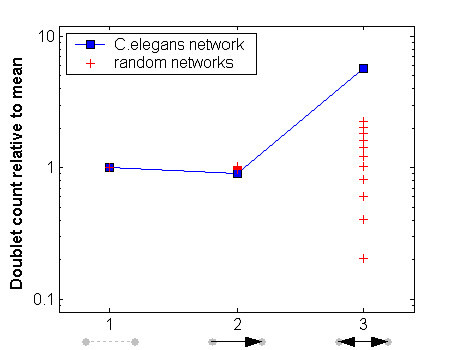
**Doublet counts in the *C. elegans *network compared to the random matrix ensemble. **Bi-directionally connected doublets are over-represented in the *C. elegans *network. Counts shown are for dataset 1, threshold Θ = 3, number of random matrices *n *= 1000. Other datasets and thresholds give similar results.

We classify all possible doublets (or pairs) of the *C. elegans *neurons into three classes: unconnected, uni-directionally connected and bi-directionally connected, and compare the number of doublets in each class to that expected in a random network (Figure 1). The random network ensemble consists of connectivity matrices that preserve the numbers of incoming and outgoing synapses for each neuron but not the identities of the partners [9,10]. The motivation behind this choice of the random matrix ensemble and the details of the algorithm are explained in Methods.

We find that the number of doublets in each class deviates from the mean of the random matrix counts, as shown in Figure 1 for a representative threshold Θ = 3. For the purposes of module search, the most interesting finding is the over-representation of the reciprocally connected doublets (pattern #3), for two reasons. First, if a set of neurons were to function as a module it should not consist of two (or more) disconnected subsets. This consideration rules out pattern #1. Second, since our search for modules is aimed at identifying over-represented inter-connectivity patterns we are less interested in under-represented ones. This consideration rules out pattern #2. We note that pattern counts are not independent, but are subject to sum rules. For example, the number of neurons in the network fixes the total doublet count. Also, the total number of connections is equal to the count of pattern #2 plus twice the count of pattern #3. These sum rules place stringent constraints on possible combinations of doublet counts. Yet, for patterns with greater number of neurons (*N>2*), these constraints become less stringent because the number of patterns increases (see below).

We repeat the above calculations for other datasets and threshold values and consistently find the significant over-representation of bi-directionally connected doublets (data not shown). In *C. elegans*, such over-representation was reported previously on a qualitative level [4]. Interestingly, an over-representation of bi-directionally connected doublets was also found for pyramidal neurons in mammalian neocortex [11-13]. This suggests that motifs may represent evolutionary conservation or convergence driven by similar computational constraints. Next, we discuss whether *C. elegans *can provide a clue to the functional significance of the over-representation of reciprocally connected doublets.

Can bilateral (left-right) symmetry of the *C. elegans *neuronal network account for the over-representation of the reciprocally connected doublets? Indeed, about two thirds of *C. elegans *neurons have a bilaterally symmetric partner. If connections between these pairs obeyed bilateral symmetry then they could not be uni-directional, creating a bias in favor of bi-directional connections. To see whether this is the case, we calculate the percentage of bi-directional connected doublets, which consist of a bilateral neuron pair. We find that these percentages are small: 7.1% and 5.5% in datasets 1 and 2, respectively. Therefore, bilateral symmetry is not sufficient to explain the observed result.

The over-representation of reciprocally connected doublets in *C. elegans *has been explained [6] as a consequence of correlation between adjacency and connectivity of neurons. The argument is that, if there is a synapse from neuron A to neuron B, they must be adjacent. If neurons A and B are adjacent then a synapse from B to A is more likely than chance, increasing the probability of a reciprocal connection. Analysis of original EM reconstructions [4] supports this argument [6,14]. Adjacency in this case does not refer to the nearby placement of cell bodies but to the number of EM sections (divided by five) in which the processes of the two neurons are in contact [6,14].

Although correlation between adjacency and connectivity may account for the over-representation of reciprocally connected doublets, why such correlation would exist in *C. elegans *remains unclear. It could be that the number of neuronal pairs, which can be adjacent, is limited by physical constraints. This would restrict the adjacent pairs only to the ones that need to connect for functional reasons. Indeed, volume exclusion explains neuron dimensions in the cortical column ([15] and references therein). In the *C. elegans *network, however, the small number of neurons should in principle allow a contact between any pair of neurons. This argument is supported by the observation that many neuronal processes are longer than the distance between the corresponding cell bodies, suggesting that the connection can be made. However, processes tend to run in bundles and make synapses only in their (often varying) neighborhoods [14]. This suggests that other (e.g. developmental) constraints may restrict the number of adjacent neurons. Alternatively, it could be that network functionality requires over-representation of reciprocal connections (or clustering). These issues must be explored in the future.

### Several triplet classes (*N *= 3) are over-represented

**Figure 2:**
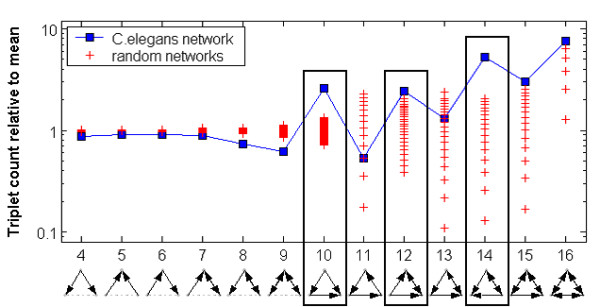
**Triplet counts in the *C. elegans *network compared to the random matrix ensemble. **Blue squares show triplet counts for the actual network (dataset 1, threshold Θ = 2), red crosses show counts for each random connectivity matrix relative to the mean count for the whole random matrix ensemble. Three framed motifs are discussed in the main text. All matrices in this ensemble (*n *= 1000) preserve the counts of uni- and bi-directional connections for each neuron. A count for a given pattern is often the same in many matrices resulting in few crosses (e.g. there are only 25 crosses for pattern 12 because the count of this pattern in the random matrix ensemble varies between 6 and 32; 29 and 31 were not observed).

We classify all connected triplets in the *C. elegans *wiring diagram into 13 classes and count the number of triplets in each class. We compare the actual number of triplets in each class to the null-hypothesis random matrix ensemble defined as follows. In order to include the observed over-representation of reciprocally connected doublets, we construct random networks that preserve the numbers of bi-directional and uni-directional connections for each neuron. Figure 2 shows triplet counts for each class relative to the mean of the random matrix ensemble. For threshold Θ = 2 we find that several triplet counts are noticeably different from the mean of the random matrix ensemble, e.g. patterns #10, #12, #14 and, possibly, #15 and/or #16 in Figure 2. Similar results were found for other values of the threshold (within the biologically plausible range, Θ = 1 to 7).

Are these differences between triplet counts in actual and random networks significant? One might answer this question by calculating, for each class, a significance *p*-value, i.e. the probability of finding a random matrix with deviation from the mean exceeding or equal to that for the actual network. Although such an approach would be correct if over-representation of a single class were examined, it would over-estimate the true significance (i.e. under-estimate the *p*-value) when many different classes are evaluated simultaneously. This situation is known as multiple hypothesis testing and requires an adjustment of the raw *p*-values (see Methods).

**Figure 3:**
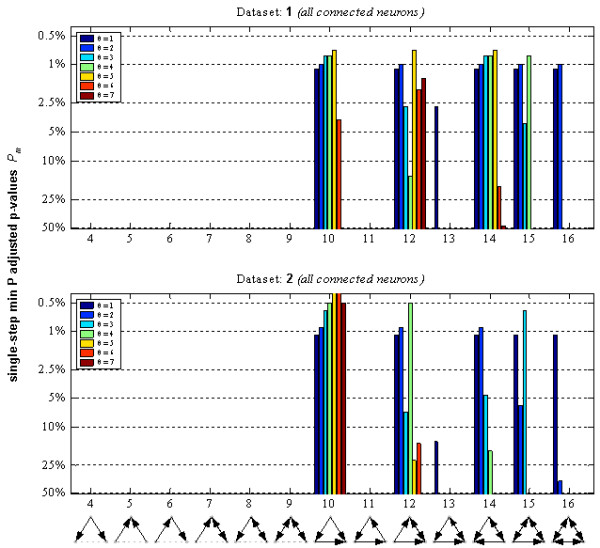
**Significance measure *P*_*m *_of triplet over-representation for different thresholds. **The multiple hypothesis testing corrected *p*-values for triplet patterns show significant over-representation of patterns 10, 12 and 14 (datasets 1 and 2, *n *= 1000). The significance measure *P*_*m *_represent the probability of finding a random matrix *R*, in which at least one class has smaller (or equal) raw *p*-value than that found for a given class in the actual network.

We chose to perform multiple hypothesis testing adjustment by controlling the family-wise error rate, i.e. the probability of mistakenly reporting at least one non-over-represented pattern, by using the *single-step min P *procedure [16,17]. The adjusted *p*-values for every class and threshold represent the probability of finding a random matrix *R*, in which at least one class *i *has smaller (or equal) raw *p*-value than that found for a given class and threshold in the actual network. This measure can be calculated by counting the number of random matrices, which have a smaller (or equal) raw *p*-value (in at least one class) than that in the actual network for a given class and threshold. By dividing this number of matrices by the total size of the random matrix ensemble, we estimate the multiple hypotheses testing corrected significance measure *P*_*m *_for each class and threshold, Figure 3 (see Methods).

According to the significance measure, *P*_*m*_, one of the most consistently over-represented motifs is the feedforward loop (triplet pattern #10), previously noticed in *C. elegans *[5,18] and other networks [7,8]. For the full list of feedforward loops see Supplementary Information [Additional files 2 and 3]. Could some known feature of neuronal organization account for the observed over-representation of the feedforward loop? We consider two hypotheses:

#### i. The three-layered feedforward neuronal network is not sufficient to account for over-representation of the feedforward loop

**Figure 4:**
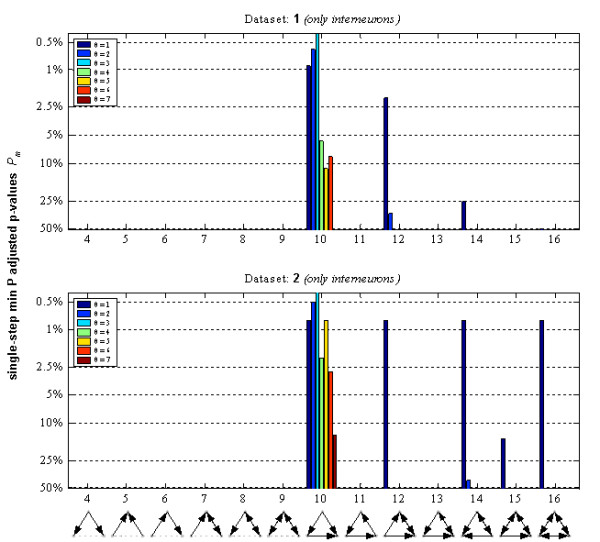
**Significance measure *P*_*m *_of triplet over-representation among interneurons shows that pattern #10 is significantly over-represented. **Multiple hypothesis testing corrected *p*-values for the triplet over-representation in datasets 1 and 2 (*n *= 1000). The figure shows that motif #10 is significantly over-represented within interneurons.

If one views the *C. elegans *nervous system as a three-layer feedforward network, where sensory neurons synapse mostly on interneurons, and interneurons synapse on other interneurons or motorneurons, this could explain the over-representation of the feedforward loop. We argue that this is not the case for two reasons. First, the feedforward loop is also over-represented among interneurons (Figure 4). Second, the three-layer model of the *C. elegans *nervous system is overly simplified. For example, there are feedback connections from interneurons to sensory neurons and from motorneurons to interneurons. To evaluate whether detected feedforward loops fit the three-layer feedforward network, we analyze the function of the neurons in these loops. About 40% of the detected feedforward loops either contain all neurons from the same functional group or at least one connection goes from a neuron in a lower layer to a neuron in a higher layer, Table 1. These loops do not fit into this three-layer model, undermining the hypothesis.

#### ii. The likelihood of connectivity between nearby neurons may partially account for over-representation of the feedforward loop

Since connectivity and adjacency are correlated in *C. elegans *and other nervous systems one could argue the following [4]. If two neurons have a common synaptic partner, then they are likely to be adjacent to that common partner, and hence to each other. If the two neurons are adjacent they are likely to be connected to each other. Again, adjacency cannot refer to the cell body position: The fraction of over-represented triplets that consist of neurons belonging to the same ganglia is typically less than 30%. Yet this argument could be valid if the adjacency refers to the contacts between neuronal processes (see above) and needs to be verified using original EM reconstructions [4]. The problem with this argument is that it would also predict an over-representation of all strongly connected patterns (#10 to #16), as opposed to the weakly connected patterns (#4 to #9). Yet, strongly connected triplet classes #13 and #11 (the feedback loop) are not over-represented (Figure 3) so further explanation is required.

It is possible that the over-representation of the feedforward loop is a consequence of other factors or their combinations (such as feedforwardness and locality of connectivity combined). But even if these factors are found, the characterization of the network in terms of over-represented motifs remains valid. The over-representation of the feedforward loop still requires a functional explanation just as the bi-directionally connected doublet does. In gene transcription regulation networks, the feedforward loop was proposed to carry out information processing functions such as filtering out fluctuations and responding only to persistent stimuli [7]. Feedforward loop can also carry out other functions [5,18], depending on the polarity of synapses involved and the dynamic response of neurons. Once these factors are established experimentally, motif function can be analyzed theoretically.

In addition to the feedforward loop, we find that two other (both symmetric) patterns are consistently over-represented: pattern #12 and pattern #14 (Figure 3). For the full list of these patterns see Supplementary information [Additional files 2 and 3]. Previous work [8] did not identify these patterns as motifs because of their low absolute count at the only threshold considered (Θ = 5). Again, we ask whether this could be a consequence of the bilateral symmetry of the *C. elegans *nervous system. Indeed, the bilateral symmetry implies that pairs of bilaterally symmetric neurons are also connected symmetrically, meaning that triplets containing such a pair are likely to be symmetric. However, we find that the fraction of triplets #12 and #14 containing a bilaterally symmetric pair of neurons and an unpaired neuron is rather small (between 10% and 20% in datasets 1 and 2). This suggests that the bilateral symmetry of the nervous system is not sufficient to explain the over-representation of pattern #12 and #14.

Just like in any other screening algorithm, our criteria for outliers are somewhat subjective and the goal is to draw attention to interesting candidates. We limit our discussion to over-represented patterns #10, #12 and #14 because in our judgment they are most robust outliers based on the several criteria used. The reader may judge that some other patterns are over-represented as well. For example, patterns #15 and #16 are significantly over-represented for small thresholds (Figure 3). Because the absolute counts of these patterns in the *C. elegans *network are small, we cannot verify that they are consistently over-represented. Further work on larger datasets will show whether these patterns may be viewed as motifs.

### Several quadruplet classes (*N *= 4) are over-represented

**Figure 5:**
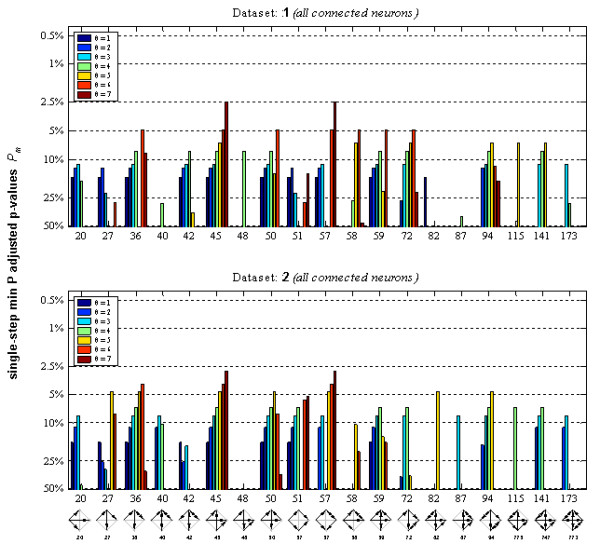
**Significance measure *P*_*m *_of selected quadruplet over-representation for different thresholds. **Multiple hypothesis testing corrected *p*-values for the quadruplet over-representation in datasets 1 and 2 (*n *= 1000). The patterns shown satisfy the following selection criteria: There must be a significant value *P*_*m *_< 0.1 for at least one Θ and the count of this pattern in *C. elegans *must be at least 5. All random matrices in the ensemble (*n *= 1000) preserve the number of uni- and bi-directional connections for each neuron as well as the count in all triplet classes for the whole network.

We classify all connected quadruplets into 199 classes and count the number of quadruplets in each class. Then we compare the actual counts of quadruplets in each class to the mean counts of quadruplets in a random matrix ensemble. In this case, random matrices preserve the numbers of uni-directional and bi-directional connections for each neuron and, in addition, the numbers of triplets (see Methods). Because of the large number of quadruplet classes, we show results (Figure 5) only for patterns selected according to the following criteria: the multiple hypothesis testing corrected significance values *P*_*m *_must be less than 0.1 for at least one threshold per pattern, while the number of quadruplets in the actual network must be at least 5. The last condition excludes patterns that may appear as over-represented due to very small quadruplet counts.

We find that quadruplet pattern #45 is consistently over-represented [8]. Can we explain this observation by some other known factor? We consider the following two hypotheses:

#### i. Bilateral symmetry of the nervous system is not sufficient to explain the over-representation of the quadruplet pattern #45

**Figure 6:**
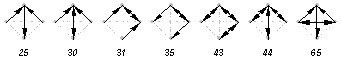
Examples of symmetric quadruplet patterns that are not over-represented.

One could propose that symmetric patterns should be over-represented because of the bilateral symmetry of the nervous system. We think that this argument by itself cannot explain the observed over-representation for two reasons. First, the fraction of feedforward quadruplets containing two bilaterally symmetric neuron pairs in motif 45 is rather small (less than 10% in dataset 1 and less than 14.3% in dataset 2). Second, many symmetric patterns are not over-represented, such as, for example, patterns 25, 30, 31, 35, 43, 44 and 65 (Figure 6).

#### ii. Feedforward structure of the nervous system may partially explain the over-representation of the feedforward quadruplet

One could propose that the feedforward three-layer structure of the nervous system could account for this observation (see over-represented triplets). We find that 14% to 37% of the feedforward quadruplets do not fit into this proposition because either they contain a feedback connection or all neurons belong to the same layer (Table 2). After comparing these percentages to the relative excess values we conclude that the feedforward structure may explain over-representation for some threshold values but not for others.

It is possible that some other factors (in addition to feedforwardness) account for the reported quadruplet over-representation. Just as argued in case of triplets, discovering these factors would be complementary to the characterization of the over-represented motif. It would be particularly interesting to determine the functional role of these motifs. Again, we arbitrarily limit our discussion of over-represented quadruplets to pattern #45. The reader may judge that some other patterns are over-represented and deserve attention (e.g. patterns #36, 50). This is why in Figure 5 we show all the outliers satisfying relatively weak criteria.

### We find no over-represented quintuplet classes (N = 5)

We classify all connected quintuplets into 9364 classes (out of 9608 patterns total, i.e. connected and unconnected) and count the actual number of quintuplets in each class. We compare these counts with the mean of the random matrix ensemble. In this case, the random matrices preserve the numbers of uni- and bi-directional connections for each neuron and, in addition, keep the numbers of all triplets and quadruplets in a 10% range of the actual network. We do not find any significantly over-represented quintuplets. This could happen because there are no significantly over-represented quintuplets with a given number of quadruplets. Alternatively, this could happen because specifying the numbers of triplets and quadruplets constrains the number of quintuplets in any random matrix the size of the *C. elegans *network. Therefore, absence of significantly over-represented quintuplets in *C. elegans *does not rule out the existence of five-neuron modules that can be detected as motifs by applying our algorithm to larger networks.

## Discussion

By comparing counts of multi-neuron patterns in the *C. elegans *wiring diagram to the mean counts of the appropriate random matrix ensemble, we find several over-represented motifs. First, we find that bi-directionally connected doublets (out of three possible doublet classes) are over-represented, given the number of connections on each neuron is fixed. Second, several triplet classes (out of thirteen possible connected patterns) are over-represented, given the actual number of bi-directional (as well as uni-directional) connections for each neuron. Third, we find that several quadruplet classes (out of 199 connected patterns) are over-represented, given the numbers of triplets are preserved in addition to previously listed constraints. We find no over-represented quintuplet classes. Some of these results, such as the over-representation of the feedforward loop and the feedforward quadruplet, have been reported previously [5,8,18]. The current paper extends and complements previous reports by performing a systematic motif search for various connection multiplicity thresholds and rigorous statistical significance assessment. Also, we consider whether the discovered motifs can be accounted for by previously known facts about the organization of the nervous system. There is no functional explanation for the existence of the motifs. Therefore, the identified motifs are candidates for modules that may perform stereotypical functions in the *C. elegans *nervous system, and they need to be investigated further.

Although the main motivation for this work, search for modules, led to our focus on over-represented patterns, we also checked for under-representation. For example, previous work indicated that the number of triplets with pattern #11 (or feedback loops) was small [6]. To determine significance, we applied the *single-step min P *procedure to the absolute value of the deviation of counts from the mean. We found that the feedforward loop is not significantly under-represented, yet many other patterns, such as weakly connected triplets were significantly under-represented (see Supplementary Information [Additional File 1]).

Our motif search algorithm is different from previous attempts to find modules [19]. For example, traditional clustering approaches look for the subsets of nodes, which are connected with their own subset more strongly than with other subsets. In our algorithm, we consider all the connections within a pattern (unlike [20], who considered only some connections within the pattern) but ignore the connections with neurons outside the pattern. One could question the expediency of ignoring multiple possible inputs to the neurons in a module since those inputs could influence the operation of that module. To counter this, we point out that if there were a particularly recurring way to attach an external connection to a given *N*-neuron motif then it would appear as an *N *+ 1-neuron motif. If, on the other hand, the motif is connected in many different ways in different instances, their significance will be washed out. Thus our approach may hierarchically detect modules with recurring input/output sites, growing them out of smaller patterns. A second justification for looking at *N*-neuron patterns is that the nervous system is capable of performing many different functions under different circumstances and neurons active in one case may be silent in another. Therefore, in any particular case, many of the anatomical inputs to the module may remain silent and can safely be ignored. This speculation may be verified experimentally by simultaneous monitoring of neuronal activity in different neurons.

The strategy and algorithms we described in this paper can be applied to incompletely mapped networks because a highly significant pattern is also likely to be over-represented in a sub-network. However, the statistical power of our algorithm increases with the knowledge of the wiring diagram. Therefore it was natural to choose the *C. elegans *nervous system, whose wiring diagram is largely known. Unfortunately, *C. elegans *has some disadvantages when it comes to the interpretation of the results: the polarity of a synapse (excitatory vs. inhibitory) in *C. elegans *is often unknown; electrophysiological investigations are still difficult in *C. elegans *[21]; and the whole network contains only 302 neurons, limiting the statistical power of the approach. Yet we hope that recent technological developments [22] will eliminate the first two disadvantages and allow functional analysis of the discovered modules. Moreover, we expect that our results have implications for understanding nervous system structure and function beyond *C. elegans*. The modules we identify in *C. elegans *may be a general property of the nervous system, and, once identified, can be searched for in other species. Finally, the algorithm itself can be applied to other networks [8] once they become available.

As in any other theoretical analysis, we made several simplifications. For example, we assumed that the strength of synaptic connection between a pair of neurons is characterized by its multiplicity (i.e. the number of synapses between that pair). This assumption may be questioned if synapses implementing high-multiplicity connections are weaker than those implementing low-multiplicity connections, as known to happen in nematodes [23]. Yet, this assumption represents a reasonable first step in the systematic quantitative analysis, which may be extended in the future by estimating synaptic strength from the original EM reconstructions. In addition, we ignored the polarity of the synapses and the existence of gap junctions. Yet our results are robust to the inclusion of these factors in the future because if an over-represented class is found, it will remain over-represented even if divided into smaller sub-classes. It would be interesting to see whether the inclusion of the above factors will reveal specific over-represented sub-classes.

## Conclusions

We have shown that certain neuronal connectivity patterns are significantly over-represented in the *C. elegans *nervous system. These patterns, called motifs, are candidates for computational modules that may perform stereotypical functions. It would be interesting to determine what these functions are and whether these motifs appear in other nervous systems.

## Methods

### Representation of the networks

We transformed the *C. elegans *synaptic connectivity data into a binary matrix *A*, called *Adjacency Matrix *or *Connectivity Matrix*, in which an entry *A*_*ij *_is 1 if there is a connection from neuron *i *to neuron *j *and 0 otherwise. The order in which neurons are assigned to rows in this matrix is not important for our calculations. The multiplicity of synapses between two given neurons is mapped to a binary value by applying a threshold to the data. We assume a synaptic connection of threshold Θ from neuron *i *to neuron *j *if neuron *i *makes at least Θ synapses on to neuron *j*. Adjacency matrices that we used are available in the Supplementary Information [Additional files 2 and 3].

### Detecting & counting patterns

We implemented two strategies for counting the number of triplets, quadruplets and quintuplets in a given connectivity matrix. First, to obtain the count of all *N*-neuron patterns, we took all different *N*-neuron subsets and characterized their connectivity. Second, we took all possible *N*-neuron subsets out of the neighborhood of a neuron *x*. This neighborhood is defined by all neurons that can be reached from *x*, if the directed connectivity matrix is made undirected. In both cases it is crucial for the run time of the algorithm to detect the pattern class from these connectivity sub-matrices as quickly as possible. We realized this by defining a function that maps each possible *N*-neuron sub-matrix to a unique integer value. Then we classified all the sub-matrices based on the function value and a pre-calculated lookup table, which identifies the pattern class from the function value.

### Creating random matrices

The number of neurons that receive synaptic input from a given neuron *x *is called out-degree of *x*. The number of neurons providing synaptic input to neuron *x *is called in-degree of *x*. In the binary matrix representation of a network as described above, the out-degree of a neuron *x *can be calculated as the sum of row *x*, the in-degree as the sum of column *x*.

**Figure 7:**
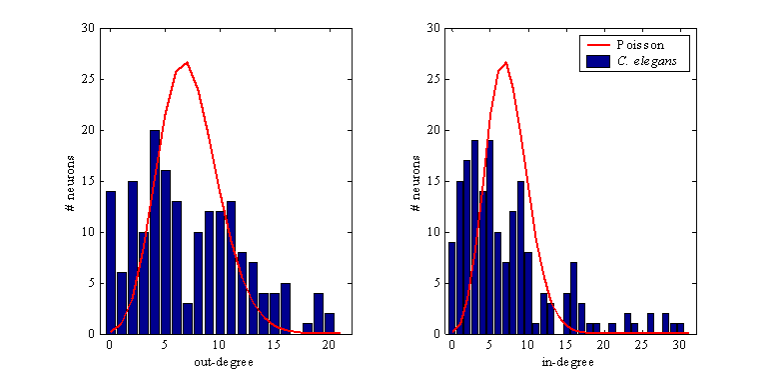
**Distribution of degree in *C. elegans *in comparison to Poisson distribution. **The distribution of in-degree and out-degree in a random matrix (Erdõs-Rényi random graph) can be approximated by Poisson. We observe that the distribution in *C. elegans *is significantly different from Poisson (p < 7%). Figure shows in-degree and out-degree for dataset 1, Θ = 1; Dataset 2 and other thresholds give similar results.

N = 2. For the first step of our analysis we create random matrices that preserve the in-degree and out-degree of every neuron but change their connection partners. Starting with an empty matrix, our algorithm selects neurons in a random order and connects each with the required number of other neurons, chosen randomly out of the remaining neurons with in-degree and out-degree less than that in the *C. elegans *network. This choice of random matrices is motivated by the observation that the distribution of in-degrees and out-degrees in *C. elegans *is significantly different from Poisson, which is expected for a randomly generated matrix without any correlations (Erdõs-Rényi random graph) (Figure 7).

N = 3. We keep the number of incoming and outgoing uni-directional connections as well as the number of reciprocal connections for each neuron the same. One of the implemented algorithms starts with an empty matrix. Then it randomly selects a neuron and does three things. It reconnects all outgoing connections of that neuron to other neurons, as long as their in-degree does not exceed that in the *C. elegans *network. It reconnects all incoming connections of that neuron to other neurons, as long as their out-degree does not exceed that in the *C. elegans *network. It reconnects all reciprocal connections of that neuron to other neurons with available unconnected reciprocal connections. We also implemented a second algorithm to verify the robustness of our results. This algorithm [9,10] will randomly pick and swap 2 unidirectional or 2 bi-directional connections (a→b and c→d will be changed to a→d and c→b).

N = 4. For comparing the count of quadruplets, we construct random matrices that keep the same not only in-degree and out-degree of uni-directional and bi-directional connections for each neuron but also the count of the 16 different 3-neuron pattern in the whole matrix. Starting from a random matrix for *N *= 3 as described above, we use the Simulated Annealing algorithm [24] by swapping two connections of the same type until the count for all triplets in the random matrix matches the real network. Since this swapping operation does not change the degrees of the various connection types for the neuron, the algorithm only has to check if the triplet count in all 16 classes is identical to *C. elegans*.

N = 5. For the analysis of the quintuplets, we modified the Simulated Annealing algorithm to match the count of all 4-neuron patterns to the real network. With this algorithm we could only find random matrices for which the relative difference between the count of each pattern in the random matrix and the real dataset was less than 10%.

### Coin-tossing example of multiple hypothesis testing correction

Here we illustrate the issue of multiple hypothesis testing by considering a classical coin-tossing example. Imagine determining whether a given coin is fair (i.e. yielding heads with probability 1/2) or not by tossing it 100 times and recording the number of heads. If the number of heads is not too different from 50, we expect that the coin is fair. The significance of the deviation in the number of heads from 50 is characterized by the *p*-value, which is the probability that a fair coin would have that or greater deviation. For example, the probability of getting 62 or more heads is about 1% and the corresponding *p*-value = 0.01. Now consider testing simultaneously 100 different coins by tossing each 100 times. Analyzing these 100 experiments for outliers reveals that one coin yielded 62 heads. Does this mean that this specific coin is unfair? Not necessarily. Even if all the coins are fair, such a seemingly unlikely result will be observed approximately once when examining 100 coins. In other words, the *p*-value estimated for a single coin is an underestimation of the true *p*-value when 100 coins are examined simultaneously. This situation is called multiple hypotheses testing and requires a modification of the *p*-value definition.

### *p*-Value calculation/multi hypotheses testing correction

Assume the number of *N*-neuron patterns in the *i*-th class in the actual network *A *and a random network *R *is given by: *c*_*N*,*i *_(*A*) and *c*_*N*,*i *_(*R*). Then the raw *p*-value is defined by:

*p*_*i *_= Pr(*c*_*N*,*i *_(*R*_*k*_) ≥ *c*_*N*,*i *_(*A*), *R*_*k *_∈ {*R*}).

Because we look for over-representation of all connected patterns in parallel (and there are *m *= 13 patterns for *N *= 3, *m *= 199 patterns for *N *= 4 and *m *= 9364 patterns for *N *= 5), there is an increased probability of finding an over-represented pattern by chance. We correct for that by calculating a multiple hypothesis testing corrected *p*-value for each pattern and threshold. This *p*-value, *P*_*m*_, reflects the probability that one random matrix *R*_*k*0 _out of our random matrix ensemble {*R*} will have at least one pattern, *i*, which has smaller (or equal) raw *p*-value than the given pattern in *C. elegans*. This is known as the *single-step min P *procedure and controls for family-wide error rate [16,17]. In mathematical notation the *single-step min P adjusted p-*values are defined by:


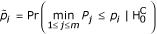


where 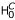 denotes the complete null hypothesis, *p*_*i *_the probability that the count for pattern *i *in a random matrix *R *is greater than the count in *C. elegans*, and *P*_*j *_denotes the raw p-value for the *i*th pattern in a random matrix *k*_0_: *P*_*j *_= Pr(*c*_*N*,*j *_(*R*) ≥ *c*_*N*,*j *_(*R*_*k*0_)).

To determine *P*_*m *_for a pattern *i *we perform the following procedure:

1. For all random matrices 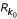 (*k*_0 _is the index of the random matrix; we usually created *n *= 1000 of them) out of the ensemble we calculate 
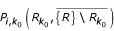
 between 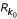 and all other random matrices in this ensemble for each pattern *i*:


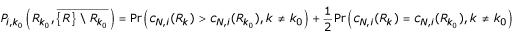
.

2. We then derive the raw *p*-value for 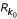 as a minimum of these values across all patterns *i*: 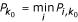.

3. We calculate the probability that for a given pattern *i *the observed count in a random matrix *R*_*k *_out of our ensemble {*R*} is greater than the count in the *C. elegans *network :


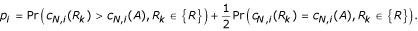


4. Last, we calculate the *single-step min P adjusted p-*value *P*_*m *_for a given pattern *i *as:


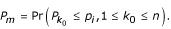


In addition, we verified our results with the alternative *single-step max T adjusted p-value *[16,17] (for figures and explanations see Supplementary Information [Additional file 1]).

### Datasets/data sources

We used data from [6], which provides separate connectivity data for the different reconstructions JSH and N2U done by White et al. (1986). We deleted 11 non-neuronal cell or classes from the dataset: CEPshDR, CEPshVL, CEPshVR, GLRDL, GLRDR, GLRL, GLRR, GLRVL, GLRVR, hyp, mu_bod. The classification of the neurons into their function and their location was taken from [20].

## Supplementary Material

Additional File 1 A document containing supplementary information and data not presented in the paper. See also Click here for file

Additional File 2 Description of the files containing triplet lists and used data sets. See also Click here for file

Additional File 3 Zip file containing files mentioned in Additional file 2 Click here for file

## Figures and Tables

**Table 1:** Feedforward loops that do not fit into consideration of a 3-layer feedforward network.

	Theta =					
		**1**	**2**	**3**	**4**	**5**
Dataset	**1**	49 %	39 %	33 %	38 %	34 %
	**2**	47 %	40 %	41 %	39 %	29 %

The table shows the percentages of feedforward loops in which all three neurons belong to the same functional group or at least one of the three connections is made from an interneuron to a sensory neuron or from a motor neuron to an interneuron.

**Table 2:** Percentage of quadruplets in pattern #45, which do not fit into the three-layer feedforward network model.

motif i = 45	Theta =					
		**1**	**2**	**3**	**4**	**5**
Dataset	**1**	37 %	31 %	29 %	25 %	23 %
	**2**	34 %	25 %	16 %	19 %	14 %

These quadruplets contain either all four neurons from the same layer or at least one connection from a motor neuron to an interneuron or from an interneuron to a sensory neuron.

**Table 3:** Number of connected neurons and the count of the different connection types after applying thresholds to the two datasets.

Source	Dataset	Threshold Θ =	Connected neurons	Total connections	uni-directional connections	Reciprocal connections
Durbin JSH	1	1	179	1152	1011	141
		2	175	649	603	46
		3	170	463	435	28
		4	161	328	313	15
		5	140	226	216	10
		6	119	163	156	7
		7	106	124	119	5
Durbin N2U	2	1	187	1288	1143	145
		2	181	728	685	43
		3	172	460	445	15
		4	162	323	313	10
		5	138	206	200	6
		6	119	143	138	5
		7	97	104	101	3
