# Supplementary material for: Search for computational modules in the C. elegans brain
Source: BMC Biol. 2004 Dec 2;2:25. doi: 10.1186/1741-7007-2-25 (PMC539283; doi:10.1186/1741-7007-2-25)
Supplement: Additional File 1 — A document containing supplementary information and data not presented in the paper. See also [file 1741-7007-2-25-S1.doc]

## Additional Information

### General

Figure 1: Distribution of the total degree (sum of in-degree and out-degree) among neurons for different thresholds in datasets 1 and 2 (blue) versus the Poisson distribution (red) that is expected for a random network (Erdős-Rényi random graph). This figure shows significant difference in the distributions for small thresholds.

### Doublets (*N=2*)

Figure 2: catalog of duplet classes used in our calculations.

Table 1: Doublet count in the actual *C. elegans* network *A* in relation to the mean count and standard deviation of the random matrix ensemble *{R}* (*n*=1000). Gray row highlights the data presented in the paper.

Table 2: Absolute number and percentage of bi-directional connected doublets consisting of a bilateral symmetric neuron pair (like e.g. AVAL and AVAR) for all different datasets and thresholds *Θ*.

### Triplets (*N=3*)

Figure 3: catalog of triplet classes used in our calculations. Motifs 1 to 3 correspond to 2-neuron motifs; they are not connected, i.e. there are isolated neurons in these motifs.

Figure 4: Two-tailed significance of triplet under-representation & over-representation. Multiple hypothesis testing corrected *p*-values for the deviation from the mean (*n*=1000). To suppress motifs with a rare appearance we calculated *P_m_* values only if the count *c_3,i_(R)* of this triplet in the random matrices was at least 5.

Table 3: We were interested in how the symmetry influences the count of triplets. We mirrored triplets with the following procedure: for each triplet we exchanged all neurons with their bilateral partner (if possible) and searched if this constructed triplet was also present in the list of triplets we found for that dataset and theta.

Table 4: Count of triplets in motifs 12 and 14, in which the bi-directional connected neuron pair *a* and *b* consists of a bilateral symmetric pair.

Table 5: Count of triplets in motif 12 and 14 that have a mirrored triplet in the same dataset/theta.

Figure 5: Significance of triplet over-representation in datasets 1 and 2, if all synapses and half of the joint synapses are taken to threshold connectivity. Multiple hypothesis testing corrected *p*-values for the triplet over-representation (*n*=1000). To suppress motifs with a rare appearance we calculated *P_m_* values only if the count *c_3,i_(R)* of this triplet in the random matrices was at least 5.

### Quadruplets (*N=4*)

Figure 6: Catalog of all quadruplet classes used in our calculations. Motifs 1 to 16 correspond to triplets, motifs 17 to 19 are not connected, i.e. there is no undirected connection between all neurons of these motifs.

Table 6: Percentage of quadruplets in motifs 45 and 51 that do not fit into 3 layer consideration, which means that all 4 neurons are in the same layer or at least one connection is from a motor-neuron to an inter-neuron or from a inter-neuron to a sensory neuron.

Table 7: Percentage of quadruplets which have bilateral symmetric neuron pairs. Motif 45: the neuron pair a, b as well as c, d consists of a left-right pair. Motif 51: just a, b consists of a left-right pair.

Table 8: Percentage of quadruplets for which the mirrored quadruplet is also present in *C. elegans.*

### Quintuplets (*N=5*)

Figure 7: List of all unconnected quintuplet classes, in which all neurons have at least degree 1. The motifs with index 1 to 218 correspond to quadruplet classes. (List of all 9608 motif pictures upon request ☺)
