# Supplementary material for: Search for computational modules in the C. elegans brain
Source: BMC Biol. 2004 Dec 2;2:25. doi: 10.1186/1741-7007-2-25 (PMC539283; doi:10.1186/1741-7007-2-25)
Supplement: Additional File 2 — Description of the files containing triplet lists and used data sets. See also [file 1741-7007-2-25-S2.doc]

# Supplementary Information for paper "Search for computational modules in the C. elegans brain"

## Triplet lists

The following table contains names of CSV files. These files contain lists of all triplets we found for the 13 different connected patterns. The lists can be downloaded with neuron numbers, neuron names or the complete connectivity information within the triplets. Neuron numbers can be transformed to neuron names with the files in the 1st column (dataset).

| **data set** **[[1]](#endnote-2)** | **triplet pattern [[2]](#endnote-3)** | **count triplets** | **list with  neuron numbers** | **list with  neuron names** | **connectivity matrices [[3]](#endnote-4)** |
| --- | --- | --- | --- | --- | --- |
| [1](http://www.cshl.edu/labs/mitya/Celegans/lists/Neurons_M2sS.csv)  List and order  of neuron names  Neurons_M2sS.csv | 4 | 3113 | iList_M2sS_i04.csv | nList_M2sS_i04.csv | mList_M2sS_i04.csv |
|  | 5 | 4469 | iList_M2sS_i05.csv | nList_M2sS_i05.csv | mList_M2sS_i05.csv |
|  | 6 | 3568 | iList_M2sS_i06.csv | nList_M2sS_i06.csv | mList_M2sS_i06.csv |
|  | 7 | 1497 | iList_M2sS_i07.csv | nList_M2sS_i07.csv | mList_M2sS_i07.csv |
|  | 8 | 1362 | iList_M2sS_i08.csv | nList_M2sS_i08.csv | mList_M2sS_i08.csv |
|  | 9 | 225 | iList_M2sS_i09.csv | nList_M2sS_i09.csv | mList_M2sS_i09.csv |
|  | 10 | 747 | iList_M2sS_i10.csv | nList_M2sS_i10.csv | mList_M2sS_i10.csv |
|  | 11 | 35 | iList_M2sS_i11.csv | nList_M2sS_i11.csv | mList_M2sS_i11.csv |
|  | 12 | 135 | iList_M2sS_i12.csv | nList_M2sS_i12.csv | mList_M2sS_i12.csv |
|  | 13 | 78 | iList_M2sS_i13.csv | nList_M2sS_i13.csv | mList_M2sS_i13.csv |
|  | 14 | 186 | iList_M2sS_i14.csv | nList_M2sS_i14.csv | mList_M2sS_i14.csv |
|  | 15 | 71 | iList_M2sS_i15.csv | nList_M2sS_i15.csv | mList_M2sS_i15.csv |
|  | 16 | 17 | iList_M2sS_i16.csv | mList_M2sS_i16.csv | mList_M2sS_i16.csv |
| [2](http://www.cshl.edu/labs/mitya/Celegans/lists/Neurons_M3sS.csv)  List and order  of neuron names  Neurons_M3sS.csv | 4 | 4019 | iList_M3sS_i04.csv | nList_M3sS_i04.csv | mList_M3sS_i04.csv |
|  | 5 | 5512 | iList_M3sS_i05.csv | nList_M3sS_i05.csv | mList_M3sS_i05.csv |
|  | 6 | 4331 | iList_M3sS_i06.csv | nList_M3sS_i06.csv | mList_M3sS_i06.csv |
|  | 7 | 1521 | iList_M3sS_i07.csv | nList_M3sS_i07.csv | mList_M3sS_i07.csv |
|  | 8 | 1607 | iList_M3sS_i08.csv | nList_M3sS_i08.csv | mList_M3sS_i08.csv |
|  | 9 | 197 | iList_M3sS_i09.csv | nList_M3sS_i09.csv | mList_M3sS_i09.csv |
|  | 10 | 895 | iList_M3sS_i10.csv | nList_M3sS_i10.csv | mList_M3sS_i10.csv |
|  | 11 | 21 | iList_M3sS_i11.csv | nList_M3sS_i11.csv | mList_M3sS_i11.csv |
|  | 12 | 130 | iList_M3sS_i12.csv | nList_M3sS_i12.csv | mList_M3sS_i12.csv |
|  | 13 | 81 | iList_M3sS_i13.csv | nList_M3sS_i13.csv | mList_M3sS_i13.csv |
|  | 14 | 250 | iList_M3sS_i14.csv | nList_M3sS_i14.csv | mList_M3sS_i14.csv |
|  | 15 | 79 | iList_M3sS_i15.csv | nList_M3sS_i15.csv | mList_M3sS_i15.csv |
|  | 16 | 21 | iList_M3sS_i16.csv | mList_M3sS_i16.csv | mList_M3sS_i16.csv |

## Used data sets

The following table provides links to the data files used for the calculations in our paper. CSV are comma separated text files, the MAT-files contain the same information but can directly be opened in MatLab.

| **Data set Error: Reference source not found** | **Adjacency matrix [[4]](#endnote-5)** | **neuron index** |
| --- | --- | --- |
| 1  (send and send_joint synapses) | dataset1.csv dataset1.mat | Neurons_M2sS.csv |
| 2  (send and send_joint synapses) | dataset2.csv  dataset2.mat | Neurons_M3sS.csv |

1. Dataset 1 contains synapses from the "JSH" reconstruction of White et al. (1986). Dataset 2 contains synapses from the "N2U" reconstruction of White et al. (1986). [↑](#endnote-ref-2)
2. Triplet patterns: [
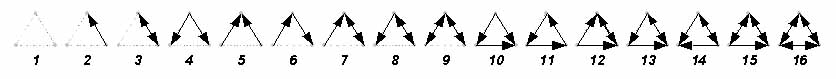
](http://www.cshl.edu/labs/mitya/Celegans/TripletClasses.jpg) [↑](#endnote-ref-3)
3. Each row of these files contains a single 3*3 sub connectivity matrix. The entries represent the number of synapses reported between the neurons. [↑](#endnote-ref-4)
4. CSV: Each row in these files defines one entry of the adjacency matrix: row index, column index and number of synapses.
   MAT: Matlab files contain a matrix "A" and vectors "Neurons" and "NeuronType". [↑](#endnote-ref-5)
